# All-Solid State Potentiometric Sensors for Desvenlafaxine Detection Using Biomimetic Imprinted Polymers as Recognition Receptors

**DOI:** 10.3390/polym14224814

**Published:** 2022-11-09

**Authors:** Majed A. Bajaber, Ayman H. Kamel

**Affiliations:** 1 Chemistry Department, Faculty of Science, King Khalid University, Abha 61413, Saudi Arabia; 2 Chemistry Department, Faculty of Science, Ain Shams University, Cairo 11566, Abbasia, Egypt; 3 Chemistry Department, College of Science, Sakhir 32038, Bahrain

**Keywords:** screen printed, single-walled carbon nanotubes (SWCNTs), solid contact, desvenlafaxine, pharmaceutical analysis

## Abstract

Using single-walled carbon nanotubes (SWCNTs) as an ion-to-electron transducer, a novel disposable all-solid-state desvenlafaxine-selective electrode based on a screen-printed carbon paste electrode was created. SWCNTs were put onto the carbon-paste electrode area, which was protected by a poly (vinyl chloride) (PVC) membrane with a desvenlafaxine-imprinted polymer serving as a recognition receptor. Electrochemical impedance spectroscopy and chronopotentiometric techniques were used to examine the electrochemical characteristics of the SWCNTs/PVC coating on the carbon screen-printed electrode. The electrode displayed a 57.2 ± 0.8 mV/decade near-Nernstian slope with a 2.0 × 10^−6^ M detection limit. In 10 mM phosphate buffer, pH 6, the ODV-selective electrodes displayed a quick reaction (5 s) and outstanding stability, repeatability, and reproducibility. The usefulness of electrodes was demonstrated in samples of ODV-containing pharmaceutical products and human urine. These electrodes have the potential to be mass produced and employed as disposable sensors for on-site testing, since they are quick, practical, and inexpensive.

## 1. Introduction

Desvenlafaxine (ODV) is an antidepressant that belongs to a class of medications known as selective serotonin and norepinephrine reuptake inhibitors (SNRI). SNRIs work by blocking the transporter reuptake proteins for important neurotransmitters that affect mood, leaving more active neurotransmitters in the synapse. Desvenlafaxine has a low affinity for muscarinic, cholinergic, histaminergic, and α1-adrenergic receptors, and inhibits serotonergic and noradrenergic reuptake receptors [1]. Monoamine oxidase inhibitory action is absent in desvenlafaxine. Through the action of Cytochrome P450 2D6 (CYP2D6) on the parent medication, venlafaxine is metabolized into O-desmethylvenlafaxine or desvenlafaxine in around 75% of cases [2]. Desvenlafaxine has a lower risk of medication interactions, notably with the CYP2D6 pathway, because it is largely removed by phase II metabolism and not the cytochrome CYP2D6 system. In addition to being researched for the treatment of vasomotor symptoms related to the menopause, ODV has been licensed for the treatment of major depressive disorder (MDD) [3,4]. Additionally, preliminary data point to the clinical utility of DVX in the management of physical discomfort and anxiety-related symptoms [5]. No pharmacopoeia recognizes desvenlafaxine succinate as an official drug. Attempts have been undertaken to develop several analytical methods for the quantification of ODV. According to a review of the literature, HPLC has been employed with UV-detection [6,7,8,9], spectrofluorimetric [10,11,12,13] or coulometric detection [14], capillary electrophoresis [15,16,17,18,19], adsorptive stripping voltammetric [20], HPLC-ESI/MS [21,22], LC-MS/MS [23,24,25,26,27], and fluorometric [28] techniques. Most of these techniques need the use of costly sophisticated equipment and challenging pre-treatment steps such as active component extraction. On the other hand, the researchers’ current objective is to create inexpensive, lightweight, portable devices that can be used in situ.

When compared with conventional methods, electrochemical sensors provide several advantages, including low instrument costs, a small sample-size need, and comparatively quick analysis times. Due to its benefits of being straightforward, quick, dependable, affordable, and nondestructive, suggesting electrochemical methods of analysis employing ion-selective sensors is an appealing and alternative way for organic and inorganic detection [29,30,31,32,33,34,35,36]. Within the literature review, only two potentiometric sensors were reported for the determination of ODV [37,38]. Such sensors’ fundamental component is the receptor, which is a molecular recognition element that can bind the analyte in a particular way. The receptor is in close contact with a transducer able to convert the analyte-recognition event into a quantifiable potentiometric signal.

Molecular imprinted polymers (MIPs) or man-tailored imprinted polymers are considered as promising and interesting materials in the field of molecular recognition. They exhibit excellent selectivity and affinity constants that are suited for sensing [39,40,41], and they are strong, stable, and resistant to degradation in acids, alkaline environments, or at high temperatures [42]. MIPs can be produced using a process called template synthesis, in which the polymerization occurs while the analyte serves as a mold molecule (template). Like the enzymatic key-lock concept, the polymerization of the functional monomer and cross-linker around the target molecule would enable the development of cavities suitable to house the target molecule itself, after the template is removed. The target molecule will then be held within the cavities in a sample when it comes into touch with the polymer, usually through weak, non-covalent interactions (Van der Waals forces, H-bridge interactions) [43]. 

In MIP-based potentiometric sensors, the polymer and transducer must be in proximity. The use of conventional electrodes necessitates a sizeable bulk. Furthermore, they are unsuitable for in situ applications because of the large number of samples and complex cell setup. The limitations of conventional electrodes have been overcome by screen-printed electrodes (SPEs), which have become excellent substitutes. A tried-and-true method for creating inexpensive, disposable, portable, and easy-to-use electrodes is screen printing. This method prints functional (pseudo)reference and counter electrodes along with the entire electrode cell on the same substrate surface [44,45]. A variety of SPE modification techniques provide a broad range of applications. Screen-printed electrodes can be modified more easily than traditional electrodes, which often require a multi-step process. For instance, the addition of metal nanoparticles, electrochemical mediators, bioreceptors, or complexing agents, might change the composition of printing inks. Additionally, the electrode surface can be altered by the deposition of polymeric or metallic coatings [46]. The development of MIP-based screen-printed electrodes has increased over the past twenty years, notably for environmental and pharmaceutical monitoring, due to the great selectivity of molecularly imprinted polymers.

The application of stress testing protocols is mandated by the International Conference on Harmonization (ICH) guidelines [47] for the identification of degradation products that may be present in drug substances and can assist in understanding the potential drug degradation route. For monitoring the desvenlafaxine drug, an all-solid-state desvenlafaxine MIP paper-based potentiometric sensor with integrated reference and working electrodes on the same slide, was integrated. The molecularly imprinted drug (MIP) beads were disseminated into a PVC membrane and used as a sensing material on top of a screen-printed platform that had been modified with conductive SWCNTs. The sensor was employed in conjunction with a reference electrode made of solid-state poly (vinyl butyral) (PVB) electrode. Impedance spectrometry, chronopotentiometry, and potentiometry were used to describe the electrochemical characteristics of the recognition sensor. The suggested sensor exhibited great selectivity, a quick and steady Nernstian potential response, and was successfully applied to the analysis of human urine and pharmaceutical formulation samples to determine the presence of desvenlafaxine. For this analyte, only two studies presented potentiometric techniques, and they created conventional carbon paste [37] and conventional membrane electrodes [38]. A comparison among these reported ISEs with the presented work is shown in Table 1. The performance characteristics of the presented sensors and their applicability to the potentiometric detection of ODV were studied in its pharmaceutical formulations and in spiked urine samples.

## 2. Materials and Methods

### 2.1. Apparatus

All potentiometric measurements were carried out using a bench pH/mV meter (PXSJ-216 INESA, Scientific Instrument Co., Ltd., Shangahi, China). The electrochemical impedance and chronopotentiometric measurements were investigated by Metrohm Auto lap (B.V., model 204, Utrecht, The Netherlands). The reference method was performed by using high-performance liquid chromatography (HPLC) coupled with UV/VIS detector (Series 200 Pump, Perkin Elmer, Waltham, MA, USA). A Millipore Milli-Q system was utilized for obtaining de-ionized water (18.2 MΩ. cm specific resistance) to prepare all solutions. All measurements were applied at room temperature (25 ± 2 °C).

### 2.2. Reagents and Materials

All used chemicals were of extra-pure analytical reagent grades. High molecular weight poly (vinyl chloride) (PVC), polyvinyl butyral (PVB), methacrylic acis (MAA), ethylene glycol dimethacrylate (EGDMA), single-walled carbon nano tubes (SWCNTs), venlafaxine hydrochloride, desvenlafaxine hydrochloride (ODV.HCl) and 2-nitrophenyl octyl ether (o-NPOE) were purchased from Sigma Aldrich (St. Louis, Mo, USA). Tetrahydrofuran (THF), benzoyl peroxide (BPO), dimethyl sulfoxide (DMSO) and methanol were obtained from Fluka AG (Buchs, Switzerland). The different dosage forms were collected from local pharmacies, which are commercially expressed as Pristiq (76 and 152 mg/tablet) for ODV.

The reference method was the reversed-phase liquid chromatographic method for the quantification of desvenlafaxine [6]. A total of 20 μL of the sample was injected into a column C_18_ (5 μm, 250 mm × 4.6 mm i.d.). The amount of ODV was determined using a UV detector operated at a wavelength of 228 nm

Stock solutions of 10^−2^ M desvenlafaxine hydrochloride were prepared by dissolving 0.38 g of the salt drug in 100 mL of double distilled water. Serial dilutions occurred to obtain different concentrations of the drug (10^−2^–10^−7^ M), using 10 mM phosphate buffer at pH 6.0. All solutions were kept at 4 °C.

### 2.3. MIPs and NIPs Synthesis 

For this investigation, the bulk polymerization technique protocol was used for the polymer’s synthesis and characterization [42,45]. A total of 20 mg of the template ODV were dissolved in 6 mL of dimethyl sulfoxide (DMSO) at 40 °C for 5 min, while being stirred during the synthesis. Following this, 2-mmol of methacrylic acid (MAA), a function monomer, was added to the mixture, and heated for an additional 10 min at 40 °C. A total of 3-mmol of ethylene glycol dimethyl acrylate (EGDMA), a cross-linker, was added to the mixture after it had been heated to 60 °C. To remove oxygen from the reaction, the system was heated for 10 min before being purged with nitrogen gas for 5 min. In a closed reaction system, 50 mg of benzoyl peroxide (BPO), an initiator, were added, and after a steady increase in temperature to 100 °C, polymerization was completed in 1 h. After being transferred to a Petri dish, the MIP was briefly dried in a 70 °C oven for a few mins. In a 50-mL centrifuge vial containing 40-mL of methanol, the MIP particles were added to the vial to remove the templet and produce the cavities. The vial was put into the shaking incubator, and this process was performed six times, while maintaining a temperature of 35 °C and 150 rpm of shaking. The mixture was centrifuged for five minutes, and the supernatant was then examined, using HPLC, for the imprint molecules. Until the imprinted molecules could no longer be found, this procedure was repeated. The NIP was similarly synthesized and processed, but there was not a templet in the reaction mixture.

### 2.4. Sensor Fabrication and EMF Measurements

**Figure 1:**
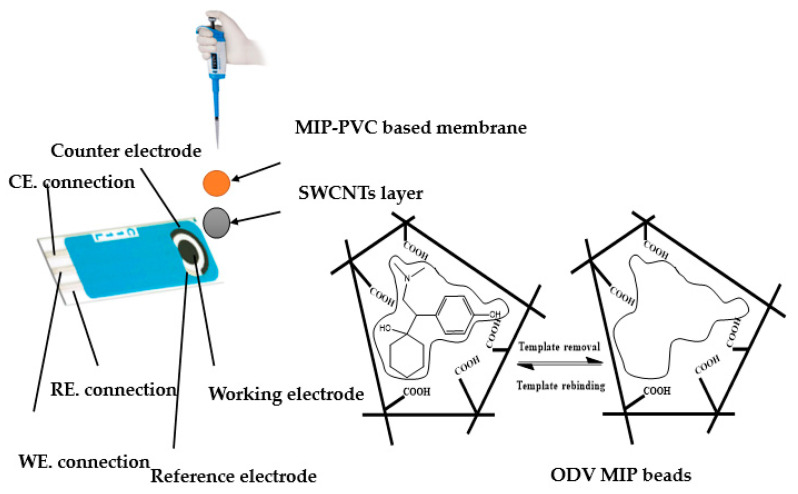
Schematic diagram for the sensor design and the pathway of the imprinting process.

Commercial screen-printed devices were used, all of them purchased from Metrohm DropSens Screen-Printed Carbon Electrodes (L33 × W10 × H0.5 mm; ref. C11L). The working (4-mm diameter) and auxiliary electrodes were made of carbon, while the reference electrode was made of silver/silver chloride. Initially, the sensing membrane was prepared after dissolving 10.0 mg of either MIPs or NIPs, 2 mg of KpClTPB, 49.0 mg of PVC, and 49.0 mg of o-NPOE in 2.0 mL THF. The working electrodes were modified by drop-casting of 10-µL of 2-mg SWCNT/1 mL THF. After complete dryness, 10-µL of the sensing membrane cocktail was drop-casted over the modified conducting carbon orifices (Figure 1). The solid-state Ag/AgCl reference electrode was fabricated after adding 10-µL of the reference membrane. This membrane consists of 75.0 mg PVB and 50.0 mg NaCl dissolved in 1 mL of methanol, which was cured at 60 °C for 30 min. After that, an insulator layer was then placed onto the printed electrodes except for the two orifices of working and reference spots and the area of the electrical contact. The fabricated electrodes were stored at 4 °C when not used and used directly in the potentiometric measurements. The fabricated electrodes were conditioned in 10^−2^ mol/L ODV solution for 2 h prior to their use. The calibration plots were constructed after plotting the potential readings versus the logarithm [ODV]. The electrodes were stored in 10^−2^ mol/L ODV solution when not in use.

### 2.5. Electrochemical Impedance and Chronopotentiometric Measurements

Electrochemical impedance spectroscopy (EIS) measurements were carried out using a three-electrode cell, including Ag/AgCl/KCl (3 mol/L) as a reference electrode (6.0729.100, Metrohm AG CH-9101 HERISAU, Switzerland) and Pt wire as an auxiliary. The practical frequency range was adjusted to between 100 kHz and 0.1 Hz, with a sinusoidal excitation signal, through an excitation amplitude of 10 mV. The measurements were applied by using a solution of 10^−3^ mol/L of ODV in phosphate buffer (10 mmol/L) of pH 6.0, at an ambient temperature of 25 ± 1 °C.

For chronopotentiometric measurements, ±1 nA current was applied to the working electrode for 60 s, and the drift in potential was recorded against the time. 

### 2.6. Analytical Applications

Drug stores provided some medications with desvenlafaxine, which is frequently used as an antidepressant. Ten pills were well ground before being sonicated in 5.0 mL of pH 6.0, 10 mM phosphate buffer. Using the developed all-solid-state ODV electrode, an aliquot equivalent to one tablet was employed for direct potentiometric measurements. A blank experiment was run in the same manner, and the recorded potential was compared with the calibration plot.

Adult males and females provided their urine samples, which were then spiked with various ODV doses and submitted to potentiometric drug assays. The goal of the experiment was to examine how a complex biological matrix might affect the performance of ODV-membrane-based sensors and drug recovery.

## 3. Results and Discussion

### 3.1. Sensors’ Characteristics

The presented all-solid-state electrodes for ODV assessment were fabricated using synthesized main-tailored MIP beads as an active and selective recognition element. The non-modified (C/MIP-ODV) and the modified (C/SWCNTs/MIP-ODV) electrodes were labeled as electrode I, and electrode II, respectively. For the membrane-based sensor (C/SWCNTs/NIP-ODV) was labelled as electrode III. The performance characteristics of the presented electrodes were presented in Table 2. For electrodes I and II, they exhibited a Nernstian cationic slope of 56.4 ± 1.1, and a 57.2 ± 0.8 mV/decade with detection limits of 3.0 × 10^−6^, and 2.0 × 10^−6^ M (*R^2^* =0.999), respectively. For electrode III, it exhibited a sub-Nernstian response with a slope of 38.3 ± 2.3 mV/decade over the linear range of 7.0 × 10^−5^–1.0 × 10^−3^ M, and a detection limit of 5.0 × 10^−5^ M.

**Figure 2:**
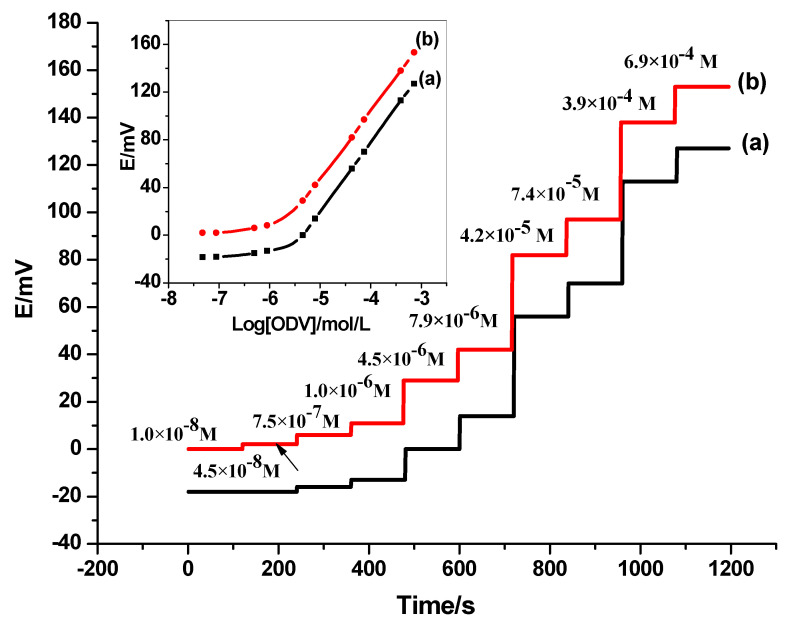
Time response of (**a**) the non-modified (C/MIP-ODV) and (**b**) the modified (C/SWCNTs/MIP-ODV) in phosphate buffer (10 mmol/L) pH = 6.0. (Inset: calibration plot.)

From the data obtained, it was found that the best analytical performances were obtained using electrodes I and II. This can be attributed to the successful imprinting of the synthesized MIP beads and proves that the solid-contact SWCNTs layer has no effect on the potential response of the fabricated electrodes. The potentiometric plots of electrodes I and II are shown in Figure 2.

### 3.2. Analytical Procedure Validation Study 

To compare a defined characteristic of the drug substance or drug product to predetermined acceptance criteria for that characteristic, an analytical technique was designed. The choice of analytical instruments and methodology must be made early in the development of a new analytical procedure, depending on the desired purpose and scope of the analytical method. Specificity, linearity, limits of detection (LOD), limits of quantitation (LOQ), range, accuracy, and precision are among the parameters that may be assessed during method development. According to ICH guidelines [47], the presented method was verified for accuracy, precision, specificity, detection limit, quantitation limit, and robustness.

#### 3.2.1. Accuracy and Precision of the Method

The recoveries of ODV using the method of standard additions were calculated to assess the method’s accuracy. A pre-quantified sample (10 µg/mL) was mixed with known concentrations of ODV (0, 4, 7.5, and 12 µg/mL), and the concentrations were then determined. The recoveries ranged from 97.8 to 103.5%. The approach is accurate, as evidenced by the high recovery-values.

By repeatedly measuring a solution containing 10 µg/mL of ODV, the instrument’s precision was assessed. Relative standard deviation was used to describe the results. The results of the intra-day and inter-day precision study of ODV are reported in terms of relative standard deviation. The study involved estimating the corresponding responses three times on the same day, and on three different days (first, second, and third day) for three different concentrations of DVX (1, 5 and 10 µg/mL). The potential measurement repeatability test was used to determine the instrument’s precision, and it revealed that the ODV’s RSD value was 1.1%. This was done to conduct intra-day and inter-day precision studies. RSD values for the ODV were determined to be 0.9–1.2% for the intra-day study and 1.1–1.5% for the inter-day precision study. The approach is precise, as evidenced by the low RSD readings.

#### 3.2.2. Detection Limit and Quantification

The lowest concentration of an analyte at which background levels may be reliably distinguished is known as the detection limit (DL). The lowest amount of analyte that can be quantitatively measured with enough precision and accuracy is the limit of quantification (LOQ) of a specific analytical method. According to ICH recommendations, DL and LOQ were determined, using the following equation:

LOD = 3.3 σ/S and LOQ = 10 σ/S

where σ is the standard deviation of the y-intercepts of regression lines and S is the slope of the calibration curve. The limit of quantification (LOQ) for ODV was 5.0 × 10^−6^ M, while the detection limit was 2.0 × 10^−6^ M. The aforementioned information demonstrates that a drug’s microgram quantity can be measured exactly and accurately. Table 2 provides an overview of the validation parameters.

### 3.3. pH Effect and Sensors’ Selectivity

**Figure 3:**
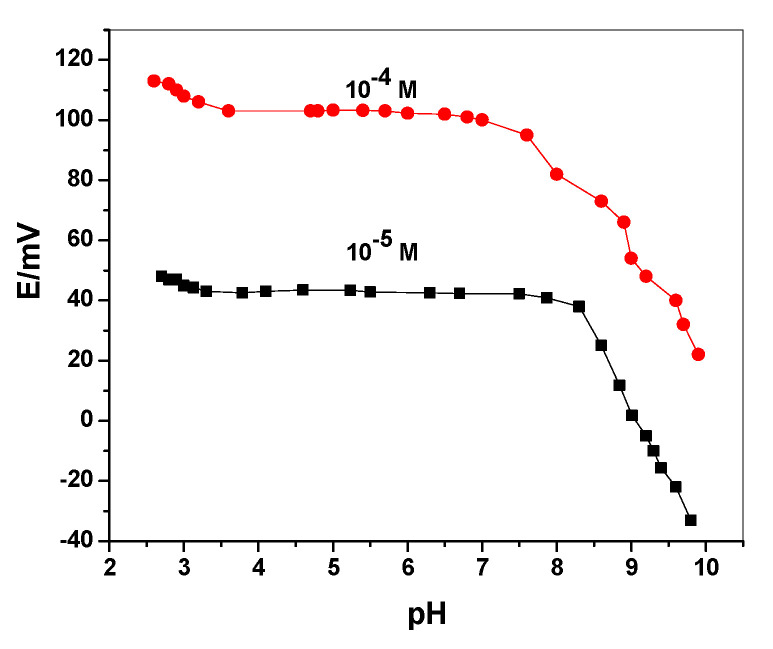
The effect of pH on the potentiometric response of C/SWCNTs/MIP-ODV sensor.

Robustness is the measure of an analytical method’s level of repeatability or reliability when subjected to deliberate modification (external factors). However, the term robustness is defined as a metric assessing the stability of the results regarding too slight changes (internal factors) [48]. To test the method’s robustness, the pH of the test solution was varied throughout a wide range, from 2 to 10. Following this, the potentiometric characteristics of an ODV membrane-based sensor were assessed at two drug concentrations (10^−4^ and 10^−5^ M), with respect to changes in pH values that were corrected using modest volumes of HCl and/or NaOH. Figure 3 depicts the effects of various pH values on the potentiometric response of the presented sensor, revealing the ranges of their stability over a pH range of 3.5–8.0 for the ODV membrane-based sensor. The pKa values of the ODV drug was 9.45 (amine) and 10.66 (phenol) [49]. At the above-mentioned pH range, ODV is completely ionized, and present in its cationic form. At pH > 8.5, the potential response dramatically declined, due to the formation of the non-cationic ODV form. Wide steady-potential reading ranges supported the investigation of highly robust and durable potentiometric sensors for ODV assessment. A phosphate buffer with a working pH of 10 mM (pH 6) was selected for additional potentiometric investigations of the applied sensor.

Investigations were conducted on the numerous interference behaviors that may manifest in the matrices during ODV determination. The modified separate solution method (MSSM) was used to evaluate the selectivity coefficient values [50]. To determine the impact of the transducer’s nature on the electrode’s selectivity behavior, the selectivity test was conducted on the presented electrodes (I and II). Since different species can coexist with the drug in either its pharmaceutical formulations or biological fluids, various interfering species were therefore chosen. These species included cationic salts (Na^+^, K^+^, Mg^2+^, and Ca^2+^), carbohydrates (glucose and lactose), medicines, amino acids (alanine, arginine, and glycine), and amino acids (tramadol, venlafaxine, and aspirin). The selectivity coefficients (*log K ^pot^_ODV,j_*) are determined in Table 3 and indicate that the suggested potentiometric sensors were not significantly affected by the contaminated interfering ions. Additionally, neither the ODV applied sensors’ selectivity behavior, nor the transducer’s type have any impact. These sensors provided highly accurate and effective ODV matrix determination. 

### 3.4. EIS and Chronopotentiometric Measurements

**Figure 4:**
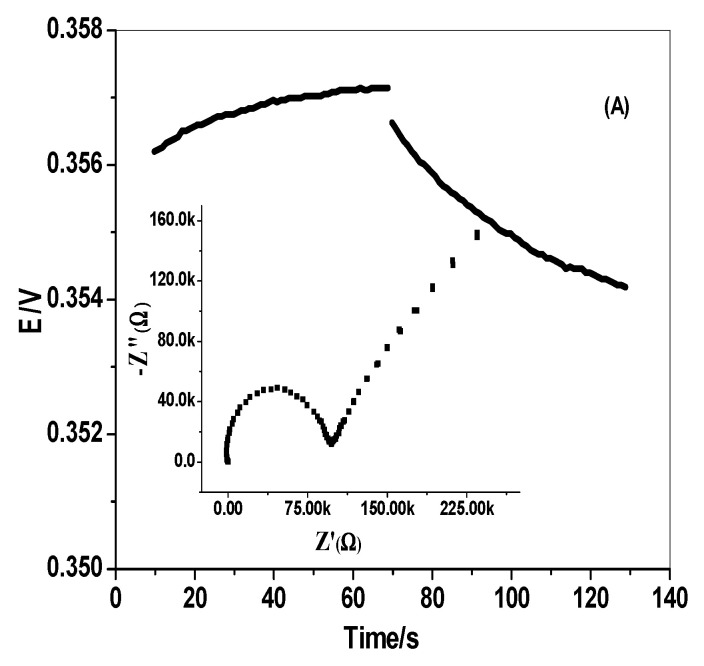

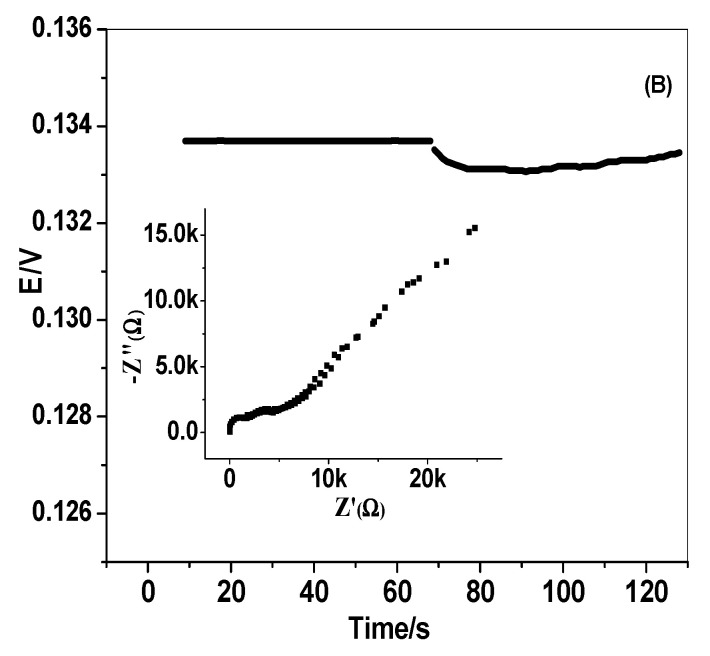
EIS and chronopotentiometric measurements for ODV membrane-based sensors. ((**A**) C/MIP-ODV, (**B**) C/SWCNTs/MIP-ODV).

As shown in Figure 4A,B, the EIS measurements revealed the Nyquist relations (complex plane plots of -Z” vs. Z^\^) on the equivalent circuit models. The unveiled results of EIS plots are represented in Table 4. The results showed the significant effect of inserting SWCNTs as a transducing material between the ion-sensing membrane and the conducting substrate. The presence of the SWCNTs layer affected the bulk resistance (R_b_*), which decreased significantly from 0.1 ± 0.04 to 0.005 ± 0.0002 MΩ. On the other hand, the existence of this transducing material increased around fivefold the double-layer capacitances (*C_dl_*) from 11.6 ± 0.6 to 91.7 ± 3.4 µF, which aided the electrical double-layer formation at the polymeric ISE membrane/solid contact interface [51]. In addition, it had a higher impact on increasing the geometric capacitances (*C_g_*), which increased from 0.03 ± 0.001 to 0.88 ± 0.06 nF. All obtained data reflect the high lipophilicity of SWCNTs and the formation of high double-layer capacitance between the ion-selective membrane (ISM) and the electronic conducting transducer.

In line with Bobacka’s method [52], the short-term potential stability of the presented electrodes was investigated. The chronopotentiograms for the presented electrodes in the absence and presence of the SWCNTs layer, are shown in Figure 4. The bulk membrane resistance (R_b_**) was calculated and decreased noticeably from 0.45 ± 0.04 to 0.15 ± 0.03 MΩ for the ODV membrane-based sensor in the absence and presence of the SWCNTs layer, respectively (Table 4). The potential drift (∆*E*/∆*t*) showed a significant decrease from 57.8 ± 1.1 to 10.6 ± 2.1 µV/s in the presence of the SWCNTs conducting layer. These data reflect the incredible increase in potential stability in the presence of the SWCNTs layer. The double-layer capacitance (*C_L_*) increased around threefold, from 13.2 ± 1.4 to 76.2 ± 1.6 µF in the presence of the SWCNTs conducting layer. The results confirm that the presence of the SWCNTs layers as transducing material between the ion-sensing membrane and the conducting substrate, exhibited high potential stability, conductivity, and high compatibility with the presented electrodes for a reliable determination of ODV in various complex matrices, without potential drift. 

### 3.5. Sensor’s Durability

**Figure 5:**
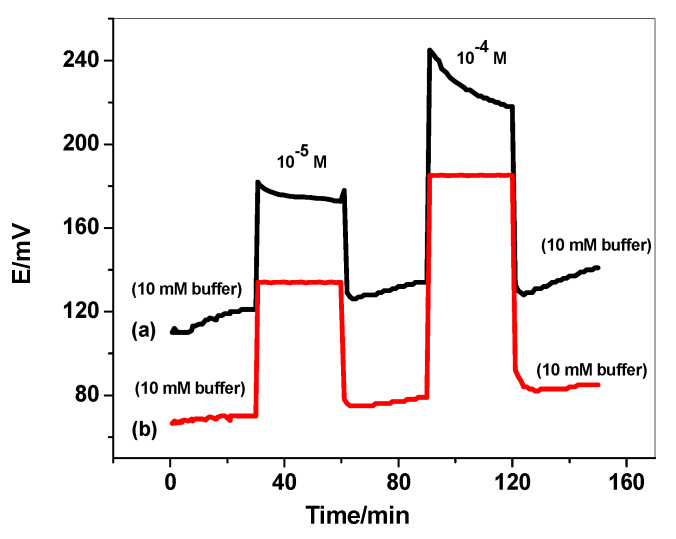
Long-term potential stability test for (**a**) C/MIP-ODV and (**b**) C/SWCNTs/MIP-ODV.

The durability of the proposed sensors for ODV determination was investigated after recording the potential (mV) vs. time (min). The test was performed by inserting the presented electrodes in 10 mM of phosphate buffer (pH 6) for 30 min. The solution was replaced by 10^−5^ M of ODV, and the electrodes were immersed in this solution for another 30 min. Afterward, the solution was replaced by the phosphate buffer solution, and the electrodes were left in this solution for another 30 min. The potential response of the electrodes was recorded over these time intervals. The same steps were repeated for 10^-4^ M ODV concentration. As shown in Figure 5, there is a noticeable potential drift for the electrode that does not contain the SWCNTs layer. This confirms the formation of a water-layer between the ion-sensing membrane and the electronic conductor substrate. The electrodes modified with SWCNTs exhibited high potential-stability over long time of the proceeding measurements. 

### 3.6. Assessment of ODV by Direct Potentiometric Measurements

To assess the suitability of the suggested sensor for drug analysis, electrode II was used to measure the amount of ODV in different pharmaceutical goods. The potential of the solution was determined after the medication tablets were crushed, ground, dissolved in phosphate buffer solution of pH 6, sonicated, and filtered. The findings showed that 99.7 ± 1.7–100.2 ± 0.9% of the nominal values were recovered (Table 5). These outcomes were contrasted, using HPLC, with calculated data from the reference technique [6]. The two approaches were compared using *F* and *t*-Student tests; however, the results showed no discernible difference between them, confirming the applicability of the suggested methods for determining ODV in its solutions.

### 3.7. ODV Recovery from Spiked Urine Samples

To use this sensor in identifying overdose patients, particularly in situations where a quick and accurate evaluation diagnosis is necessary, monitoring ODV in urine samples was also evaluated. Following the addition of known ODV concentrations to various aliquots of human urine samples, potentiometric measurements were taken, using the suggested sensor. As shown in Table 6, the data revealed an average recovery of 98.2% without any notable interferences from the species that are often present in samples of human urine-spiked medication.

**Figure 6:**
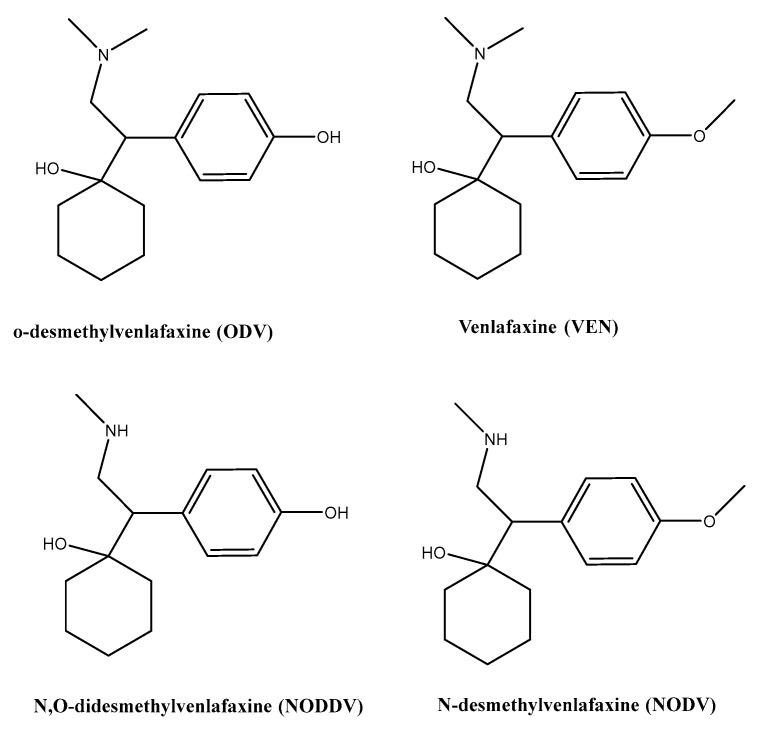
Chemical structures of venlafaxine and their metabolites.

It has been reported that desvenlafaxine is the major active metabolite of venlafaxine (VEN). VEN is highly metabolized in humans, with urinary excretion of the unchanged compound between 1–10% of an administered dose [53]. Demethylation of o-desmethylvenlafaxine (ODV), is the main metabolite produced. Approximately 45% of desvenlafaxine is excreted unchanged in urine at 72 h after oral administration. Other metabolites, N,O-didesmethylvenlafaxine (16%), and N-desmethylvenlafaxine (1%), are biologically inactive [53] (Figure 6).

The average half-life of VEN is 5.0 ± 2.0 h, whereas that of its main metabolite, ODV, is 11.0 ± 2.0 h. According to the selectivity measurements, ODV can be measured in the presence of either venlafaxine and N,O-didesmethylvenlafaxine. The metabolite N-desmethylvenlafaxine interferes during the measurements of ODV, but the amount produced is 1%. This backs up the usefulness of the sensor that was described for measuring ODV in actual samples, with little or no interference from the main drug metabolite.

## 4. Conclusions

Desvenlafaxine is monitored in some therapeutic drugs and spiked human urine samples using an affordable, miniature, all-solid state-based sensor that is very sensitive and selective in its determination. The sensor works by using MIP beads dispersed in a PVC membrane as a substance for detecting drugs. Ion-to-electron transducers and solid contact devices are both made of single-walled carbon nanotubes (SWCNTs). It considerably enhanced the sensor’s electrical characteristics and removed the influence of the water layer that existed between the selective polymeric membrane and the conductive paper substrate. The sensors revealed a near-Nernstian slope of 57.2 ± 0.8 mV/decade with the detection limits of 2.0 × 10^−6^ M (*R^2^* = 0.999), with a response time less than 8 for ODV assessment. EIS and CP measurements were used to appraise the modified sensors’ potential stability and double-layer capacities. The observable low potential drift values ((∆*E*/∆*t*) were exhibited at 10.6 ± 2.1 µV/s. These findings make the suggested strip cell design readily relevant to the pharma industry, for rapid and accurate hospital overdose-patient detection, and for potential incorporation into automated and wearable technology.

## Figures and Tables

**Table 1:** Overview on the reported potentiometric sensors of ODV determination.

Sensing Material	Electrode Type	Slope, (mV/Decade)	Detection Limit (mol/L)	Working or Linear Range (mol/L)	pH Range	Response Time, s	Ref.
Desvenlafaxine- tetraphenyl borate	carbon paste electrodes	57.9	1.5 × 10^−4^	5.6 × 10^−4^–1.0 × 10^−2^	2.0–6.0	8–14	[37]
desvenlafaxine- phosphotungstate		59.2	6.3 × 10^−5^	5.6 × 10^−4^–1.0 × 10^−2^	2.0–6.0	5–12	
Desvenlafaxine-silicomolybdate	glass tube-ISE	58.0	3.4 × 10^−6^	1.0 × 10^−5^–1.0 × 10^−2^	4–8.5	20	[38]
C/MIP-ODV	screen-printed	57.5 ± 1.2	2.1 × 10^−6^	4.5 × 10^−6^–1.0 × 10^−2^	3.5–8.0	<5	This work
C/SWCNTs/MIP-ODV		58.1 ± 1.3	1.5 × 10^−6^	4.2 × 10^−6^–1.0 ×10^−2^	3.5–8.0	<5	

**Table 2:** Analytical performance of the presented electrodes in 10 mmol/L phosphate buffer, pH 6.

Parameter	C/MIP-ODV	C/SWCNTs/MIP-ODV	C/SWCNTs/NIP-ODV
Slope(mV/decade)	56.4 ± 1.1	57.2 ± 0.8	38.3 ± 2.3
Detection limit, (M)	3.0 × 10^−6^	2.0 × 10^−6^	5.0 × 10^−5^
Correlation coefficient (*R^2^*)	0.999	0.999	0.999
Lower limit of linear range, (M)	6.0 × 10^−6^	5.0 × 10^−6^	7.0 × 10^−5^
Response time, (s)	<5	<5	<5
pH range	3.5–8.0	3.5–8.0	3.5–8.0
Life span (days)	12	15	10
Precision, (%)	1.1	0.4	1.4
Accuracy, (%)	99.2	99.5	98.3
Standard deviation, (mV)	±0.8	±0.4	±1.4

**Table 3:** Selectivity coefficients (*log K ^pot^_ODV,J_*) of the presented ODV- membrane based sensors.

Interfering Ion	*log K ^pot^_ODV,J_ ± SD* *	
	C/MIP-ODV	C/SWCNTs/MIP-ODV
K^+^	−5.1 ± 0.3	−5.15 ± 0.2
Na^+^	−5.2 ± 0.7	−5.3 ± 0.3
Mg^2+^	−5.5 ± 0.2	−5.4 ± 0.4
Ca^2+^	−4.8 ± 0.4	−4.7 ± 0.5
Arginine	−5.8 ± 0.2	−5.8 ± 0.3
Alanine	−4.3 ± 0.2	−4.4 ± 0.4
Glycine	−4.4 ± 0.1	−4.5 ± 0.3
Caffeine	−4.2 ± 0.1	−4.3 ± 0.2
Glucose	−5.5 ± 0.2	−5.6 ± 0.1
Lactose	−5.4 ± 0.3	−5.5 ± 0.2
Tramadol	−3.1 ± 0.3	−3.1 ± 0.2
Aspirin	−2.8 ± 0.1	−2.9 ± 0.3
Venlafaxine	−2.6 ± 0.3	−2.6 ± 0.1

* SD (standard deviation) for *n* = 3.

**Table 4:** Electrochemical impedance and chronopotentiometric features in absence and presence of SWCNTs.

Parameter	C/MIP-ODV	C/SWCNTs/MIP-ODV
R_b_* (MΩ)	0.1 ± 0.04	0.005 ± 0.0002
C_dl_ (µF)	11.6 ± 0.6	91.7 ± 3.4
C_g_ (nF)	0.03 ± 0.001	0.88 ± 0.06
R_b_**(MΩ)	0.45 ± 0.04	0.15 ± 0.03
∆E/∆t (µV/s)	57.8 ± 1.1	10.6 ± 2.1
C_L_ (µF)	13.2 ± 1.4	76.2 ± 1.6

**Table 5:** ODV determination in its pharmaceutical formulations using C/SWCNTs/MIP-ODV electrode.

Pharmaceutical Product	Nominal Content Taken, mg/Capsule	Found, mg/Capsule ^a^				^b^*F*-Test	*t*-Student Test
		Proposed Method	Recovery% ± SD	Reference Method [6]	Recovery% ± SD		
Pristiq^®^ (P*f*izer)	50	48.7	97.4 ± 1.1	49.7	99.4 ± 0.4	3.4	2.4
	100	103.5	103.5 ± 0.9	100.4	100.4 ± 0.2	2.3	3.1

^a^ Mean of three replicate measurements ± standard deviation (SD), ^b^ *F* and *t*-Student tests at 95% confidence level values, are 19.00 and 4.3, respectively.

**Table 6:** Recovery values for ODV assessment in spiked human urine samples.

Sample, No.	* ODV Drug, µM		Recovery, %
	Spiked	Found	
1	20.0	18.4 ± 3.4	92.0 ± 2.4
2	50.0	52.6 ± 0.4	105.2 ± 4.2
3	75.0	73.2 ± 0.4	97.6 ± 1.3

* Average of 3 measurements (*n* = 3).

## Data Availability

Not applicable.
